# Effectiveness of an online mental health strengthening module to build resilience and overcome stress for transitional aged medical students

**DOI:** 10.3389/fdgth.2023.1207583

**Published:** 2023-10-04

**Authors:** Fransiska Kaligis, Raden Irawati Ismail, Tjhin Wiguna, Sabarinah Prasetyo, Hartono Gunardi, Wresti Indriatmi, Merci Monica Pasaribu, Veranita Pandia, Kusuma Minayati, Clarissa Cita Magdalena, Garda Widhi Nurraga, Billy Pramatirta, Nicholas Calvin, Andre Sourander

**Affiliations:** ^1^ Department of Psychiatry, Faculty of Medicine Universitas Indonesia—Cipto Mangunkusumo Hospital, Jakarta, Indonesia; ^2^ Doctoral Program in Medical Sciences, Faculty of Medicine Universitas Indonesia, Jakarta, Indonesia; ^3^ Faculty of Public Health, Universitas Indonesia, Depok, Indonesia; ^4^ Department of Child Health, Faculty of Medicine Universitas Indonesia—Cipto Mangunkusumo Hospital, Jakarta, Indonesia; ^5^ Department of Dermatovenereology, Faculty of Medicine Universitas Indonesia—Cipto Mangunkusumo Hospital, Jakarta, Indonesia; ^6^ Department of Clinical Pathology, Faculty of Medicine Universitas Indonesia—Cipto Mangunkusumo Hospital, Jakarta, Indonesia; ^7^ Department of Psychiatry, Faculty of Medicine Universitas Padjajaran—Hasan Sadikin Hospital, Bandung, Indonesia; ^8^ Department of Child Psychiatry, University of Turku, Turku, Finland

**Keywords:** medical students, mental health, online module, resilience, transitional age

## Abstract

**Introduction:**

Transitional-aged youths (17-to-24-years-old) are prone to mental-health problems. Students in higher education, especially medical students, are more exposed to stressors and thus need training to increase resilience. However, there have been limited mental-health strengthening modules specifically developed for medical students of transitional age, and none in Indonesia. This study intends to test the effectiveness of an online mental-health strengthening module in altering resilience.

**Methods:**

A pragmatic randomized trial with repeated measurements was employed to evaluate biopsychosocial outcomes of resilience. The intervention module was delivered in 4 weeks to 105 eligible students. Participants were divided into intervention group (*n* = 52) and control group (*n* = 53). Outcomes were measured in the 4th, 8th, and 12th weeks. Primary outcome was resilience level as measured by Connor-Davidson Resilience Scale (CD-RISC). Perceived Stress Scale (PSS), Depression Anxiety Stress Scale (DASS) were utilized to measure stress, depression and anxiety. Knowledge and attitude toward mental-health were also measured through validated questionnaires. Stress levels of participants were measured biologically by measuring salivary cortisol and alpha-amylase levels at the baseline and 12th-week.

**Results:**

Compared to the control group, there were no significant difference in resilience score of the intervention group compared to control group [F(1, 103) = 2.243, *P* = .137]; however, there was a significant main effect of time [F(3, 309) = 18.191, *P* < .001] and interaction effect between intervention and time in resilience score [F(3, 309) = 5.056, *P* = .002]. Additionally, compared to the control group, there were significant increases in knowledge [F(1, 103) = 66.805, *P* < .001], attitudes and behavior towards mental-health [F(1, 103) = 5.191, *P* = .025], and a significant decrease in stress perception score [F(1, 103) = 27.567, *P* < .001]. The mean salivary delta cortisol during pre-test and post-test at week 12 in the intervention group showed significant difference (*P* < .001). However, there was no significant difference in the mean delta salivary alpha-amylase between pre-test and post-test at week 12, both in the intervention and control groups.

**Conclusion:**

The mental-health strengthening module was accepted and applicable to first-year medical students and was found to be effective in increasing resilience from various biopsychosocial aspects. It is also advisable to have similar modules throughout the medical school to maintain sustainability.

## 1. Introduction

Transitional-aged youth has been referred to as the population that falls under the period between late adolescents (17-to-19 years old) until early adulthood (21-to-24 years old) (1). In this transitional period to adulthood, youths may face multiple internal and external pressures. Internally, they are processing their self-image, physical and emotional changes. Externally, they might have family, peer, academic, or other life events-related conflicts (1, 2). With all these conditions, adolescents in transitional age are more likely to experience mental health problems (3). In the United States, 50 to 75% of anxiety disorders, mood disorders, and substance abuse disorders start at the age of 14 to 24 (4). Immaturity in managing emotions and solving problems might also lead youths to do high-risk behaviors such as alcohol or substance abuse, unsafe sex, or self-harm practice. Therefore, this period is a critical period for shaping youths' ability to deal with stress and the capacity to regulate emotions (2).

A common major stressor in this age group is entering college or university due to the pressure to change their ways of learning and become more self-directed (3). In Indonesia, among university students aged 16 to 24 years, more than 90% of 393 participants expressed they had financial or academic difficulties, felt lonely, and anxious (5). First-year university students stand at the intersection of physical, psychological, and environmental changes that potentially increase their stress levels (6–10). Mental health problems in university students vary between 10% to 85%, with depression and anxiety as the most common disorders reported (11). Unattended mental health problems can lead to serious consequences that affect youths' ability to live fulfilling lives in the future (12). Therefore, preventing the occurrence of serious mental health difficulties is imperative.

Resilience has been defined as a process of good adaptation to adversity, trauma, tragedy, threats, and significant sources of stress, for example, family or relationship problems, serious health problems, and work or economic environment-related stress (13, 14). Resilience is complex, multidimensional, and dynamic. It is important to note that a healthy adaptation to stress or resilience depends not only on the individual but also on the available sources of support, namely family, friends, groups, or organizations, and depends on culture, religion, community, society, and government (15, 16). Strong resilience has been linked to reduced psychological stress, increased life satisfaction, increased happiness, improved quality of life, reduced anxiety symptoms, and improved overall health (17, 18).

Resiliency training has been conducted for children and adolescents in families, schools, and community-based settings. Programs focusing on transitional youth have been developed in high-income countries and regions such as Finland, Canada, the United States, Australia, Ireland, and Asia-Pacific (19–22). However, it has not been as well researched in low-and-middle-income countries. Furthermore, in Indonesia, a country with a high population of youths, only one large-scale resiliency training program was delivered. It focused more on early adolescents in junior high schools (23). Currently, in Indonesia, no mental strengthening intervention specifically helps late adolescents face life challenges in the transition period to adulthood. Existing mental health strengthening interventions focus more on mental disorders or psychotherapy modules for specific disorders. No mental health strengthening module explicitly prepares adolescents of transition age to enter the higher education environment and the common problems often faced in such settings. With the many pressures faced by youths transitioning to adulthood in university settings, it is important to develop a resilience-based mental health training program embedded in the academic setting.

In Indonesia, medical school programs usually begin right after graduating from high school. Students in medical schools have been found to experience higher levels of stress. The resilience of medical students had been found to be lower than that of the general population in a study from Canada, stating that medical students have higher stress and lower resilience than their peers in the general population (17, 18). Thus, developing and testing the effectiveness of an online mental health strengthening module that promotes resilience in transitional-aged youth medical students is timely. To date, there is no resiliency training module specifically designed for early-year medical students in the Indonesian context. This study aimed to investigate whether a contextually adapted resiliency module delivered through online discussions and self-learning using a website can influence transitional-aged youths' resilience and mental health biopsychosocial outcomes in an Indonesian medical school setting.

## 2. Materials and methods

### 2.1. Study design

The study employed a pragmatic randomized trial design with repeated outcome measurements. Participants were divided into two groups: intervention and control groups. Outcomes for both groups were assessed at baseline, immediately at post-intervention (the 4th week), then at follow-up on the 8th and the 12th week. The study was conducted at the Faculty of Medicine, Universitas Indonesia, from August 2021 to January 2022.

### 2.2. Ethics approval

The study received full ethical approval from the Health Research Ethics Committee, Faculty of Medicine, Universitas Indonesia, with the protocol number Health Research Ethics Committee, Faculty of Medicine, University of Indonesia, numbered 20-05-0538 (KET-527/UN2.F1/ETIK/PPM.00.02/2020).

### 2.3. Sample size

The sample size was calculated to estimate the difference between the means of two independent groups (24). Based on the results of a previous resiliency-based intervention on transitional-aged youths (25), we expect a difference in means (d) of 7.6 with a standard deviation (*σ*) of 13. With a 5% level of significance and 80% power, with the formula:

$$\begin{array}{cc} & n=\frac{2\times \sigma^{2}{\left(Z\alpha +Z\beta \right)}^{2}}{d^{2}}\\ & \;n=\frac{2\times 13^{2}{\left(1.96+0.84\right)}^{2}}{7.6^{2}}\\ & \;n=45.8\approx 46 \end{array}$$

we calculated that a minimum of 46 subjects was required for each group. To anticipate the chance of a 10% drop-out, the minimum number of subjects per group was recalculated, and the minimum target was 52 subjects per group.

### 2.4. Recruitment and selection

**Figure 1:**
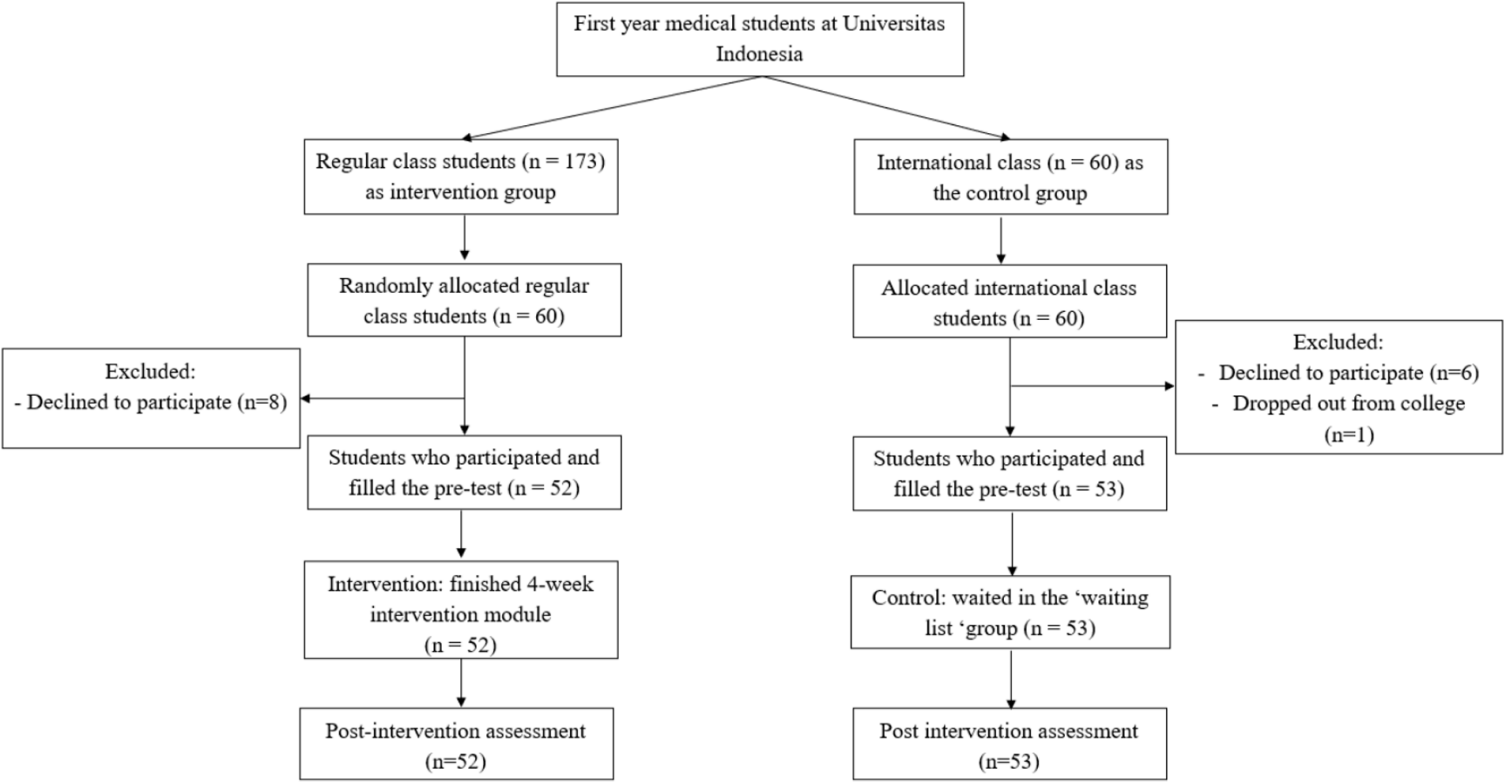
Recruitment and selection of participants.

Participants were recruited from the first-year medical students' cohort at Universitas Indonesia. The detailed recruitment process is outlined in Figure 1. There were two classes at the Faculty of Medicine: regular and international. At first, using randomizer.org, the regular class was assigned as the pool to choose participants in the intervention group, while the international class was the pool for the control group. Then, to meet the sample size, 60 students were randomly picked from the 173 students in the regular class to be recruited to join the intervention. Eight students declined; thus, 52 students consented and participated in the intervention group. All 60 students in the international class were recruited to be in the control group, but 1 student dropped out, and 6 students declined. Thus, there were 53 students consented to be in the control group. Reasons for declining include health conditions, feeling unprepared to join an additional activity, or living in another city or country with a different time zone. All other remaining students were invited to join the module after this study had finished.

### 2.5. Intervention

For the intervention group, the participants joined the “Transition and Adaptation towards Resiliency” module. The module was developed through the creation and subsequent adaptations of previous resiliency-based interventions gathered through literature review and qualitative consultations with key experts and the previous cohort of first-year medical students. The qualitative phase of the module development was intended to contextualize the intervention. The content for the module is presented on a website created specifically for the module (https://modultransisi.id/ in Indonesian, a snippet of the module in English is captured in Supplementary Material). Table 1 presents the contents of the module.

**Table 1:** Contents of the module.

Sub-module	Content
1—Changes in Life to Become More Independent and Adapting to a New Environment	Life changes in the transitional age and how to adapt to the university environment. New learning skills including time management and how to deal with exams. Introducing learning difficulties and problems that can arise in social interaction in a new environment.
2—Stress and Ways to Overcome Stress	Stress and various positive and negative strategies for dealing with stress, coping techniques for stress, encouraging students to recognize strengths, weaknesses, challenges and threats faced when dealing with stress. Introducing students to mindfulness and its related skills.
3—Mental Health Problems and Symptoms of Mental Disorders	Introducing students to mental health problems and symptoms of mental disorders. Encouraging students to recognize strengths, weaknesses, challenges, and threats faced as students at the initial level when facing mental health problems and symptoms of mental disorders, teaches students about emergency mental disorders (drug/alcohol overdose, suicide).
4—Mental Health Help-Seeking	Details the procedures for getting help for mental health problems and introduces students to places to get help on campus and in healthcare facilities. Teaches students how to help others who have suicidal thoughts.

The module intervention activities were guided online by five facilitators consulted previously during the module development. The criteria for becoming a facilitator in this module is that of a medical teacher who has been a facilitator in other learning experiences. The facilitators have been given the Module Facilitators Guidebook and access to the module website.

The module is divided into 4 submodules and delivered in 4 weeks. To complete the module, students need to participate in 8 online discussion sessions. They were required to join the online opening session (through Zoom meeting) and subsequent twice-weekly meetings.

In general, the structure and schedules of the activities of each sub-module was as follows:

1. First meeting for 90 min:
  - Explaining the general overview of the sub-module topic.
  - Explaining self-study techniques.
2. Independent learning through the website for a week.
3. Second meeting after 1 week for 90 min:
  - Reflection of participants after participating in independent learning activities in small groups.
  - Summary and reinforcement of learning objectives.

Week 1: Opening (first meeting of submodule 1), independent learning submodule 1.
Week 2: Second meeting of Submodule 1, first meeting of submodule 2, independent learning submodule 2.
Week 3: Second meeting of Submodule 2, first meeting of submodule 3, independent learning submodule 3.
Week 4: Second meeting of Submodule 3, first meeting of Submodule 4, independent learning submodule 4.
Week 5: Closing (second meeting of Submodule 4).

The opening of the module was attended by the research team, 52 students, and 5 facilitators. In the opening session, participants were introduced to the overview of the module and how to use the website for self-study by the facilitators. Then they were introduced to the first sub-module content and were divided into 5 groups for small-group discussions of the topic. The facilitators guided the small group discussions to further discuss student experiences on the topics. At the end of the session, they return to the main room to be notified about the self-study task.

The second meeting of each sub-module is held after one week of independent study activities. The activity begins with opening statements in the large group, after which students will enter small group discussions with their respective facilitators. Small group activities take place in breakout rooms in the form of self-reflective discussions and material from the website that students have studied and worked on for the previous week. The facilitators also assess student activity individually and in groups. The group discussion lasted for 45 min. The activity ended with a large group plenary for 35 min to discuss the results of each group's discussion, providing feedback from the facilitators and conclusions.

### 2.6. Measured outcomes

**Figure 2:**
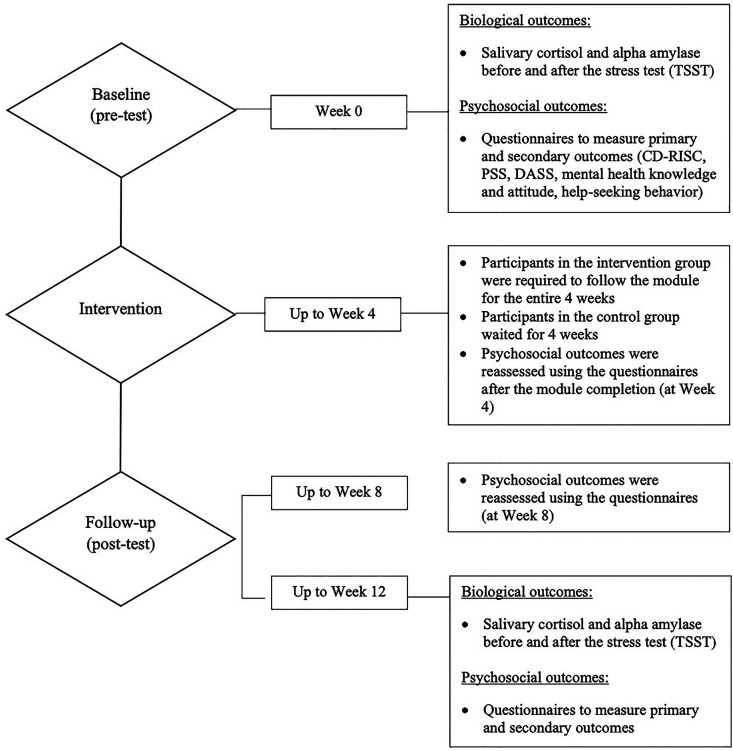
Measured outcomes timeline.

Effectiveness was investigated through pre-and-post biopsychosocial outcomes measurements. Figure 2 will elaborate when and which outcomes were assessed during the trial.

#### 2.6.1. Primary outcome measure

The Connor-Davidson Resilience Scale (CD-RISC) was utilized to measure the participants' resiliency levels (26). The CD-RISC uses a 5-point scale (0 = strongly disagree to 4 = strongly agree) and a higher score indicates a higher resiliency level. For this study, the 10-item version, which has been validated in Indonesian, was used (27).

#### 2.6.2. Secondary outcome measures

The perceived stress scale (PSS), which has been translated and validated in Indonesian, was used to measure the participants' stress perceptions in the last month (28, 29). The PSS has a 5-point scale (0 = never to 4 = very often).

To assess participants' mental health problems, the Depression Anxiety Stress Scale (DASS) was utilized to measure the participants' depression and anxiety symptoms in the past week. The 21-item version has been translated and validated previously in an Indonesian setting (30, 31). The DASS uses a 4-point scale (0 = never to 3 = very often). The depression score is obtained by adding item numbers 3, 5, 10, 13, 16, 17, and 21. The anxiety score is obtained by adding item numbers 2, 4, 7, 9, 15, 19, and 20. Meanwhile, the stress score is obtained from numbers 1, 6, 8, 11, 12, 14, and 18.

The knowledge and attitude toward mental health questionnaires translated, adapted, and validated for the study were used to measure participants' knowledge and attitude toward mental health (32).

To assess stress levels biologically, participants' salivary cortisol and alpha-amylase levels were measured at the baseline (week 0) and at the 12th-week follow-up. Cortisol and alpha-amylase were measured twice in baseline and twice in week 12 by administering a standardized stress test called the Trier Social Stress Test (TSST). Each participant was tasked to perform a presentation task in front of a scientific forum and an arithmetic task (33, 34). Before and after the TSST, the participants collected their saliva samples. The measured baseline cortisol (S1) and alpha-amylase (S1) before the TSST were compared with post-TSST cortisol (S2) and alpha-amylase (S2) to obtain a delta value (S2 – S1) for individual levels after the test.

The Trier Social Stress Test (TSST) and collection of the salivary samples were virtually directed through a video conference platform. All participants received the sampling tools and an instructional video that guides them on collecting and sending their salivary samples. Then, the TSST was conducted by presenting in front of a board of examiners and an arithmetic test for 45 min between 7 AM and 12 PM (local time) considering the natural cortisol cycle. Samples were received and analyzed by the Integrated Laboratory Installation, Clinical Pathology Department, Cipto Mangunkusumo Hospital (RSCM), Jakarta.

### 2.7. Statistical analysis

Data collected from the results of laboratory tests and questionnaires were analyzed using the SPSS version 20.0 program. Normality tests with Kolmogorov-Smirnov were conducted on all outcome measures. For normal distribution data, repeated measures ANOVA were carried out to analyze difference in mean scores between intervention group and control group across all time points (main effect), and to assess any interaction effect between within-subjects factor (time) and between-subjects factor (intervention). If the normality of data distribution was violated, a non-parametric Friedman test was carried out instead to analyze the main effect. Bonferroni post-hoc analyses between time-points would be conducted if there is significant interaction effect between time and intervention.

## 3. Results

### 3.1. Participants' characteristics and baseline measurement

In total, 105 students participated in the study. Analysis of their demographic characteristics (Table 2) revealed no significant differences between the two groups.

**Table 2:** Demographic characteristics of study participants.

Characteristics	Intervention group (*n* = 52)	Control group (*n* = 53)	*P values* (*P*)
Gender			.14^a^
Male	24 (46.15%)	17 (32.08%)	
Female	28 (53.85%)	36 (67.92%)	
Age (years)			.13^b^
15	1 (1.92%)	0 (0%)	
16	0 (0%)	1 (1.89%)	
17	8 (15.38%)	7 (13.20%)	
18	31 (59.62%)	43 (81.13%)	
19	12 (23.08%)	1 (1.89%)	
20	0 (0%)	1 (1.89%)	
High school origin (cities)			.35^a^
Jakarta, Bogor, Depok, Tangerang, Bekasi	46 (88.47%)	46 (86.79%)	
Java Island	6 (11.53%)	5 (9.44%)	
Outside of Java	0 (0%)	2 (3.77%)	
Age when starting to live without parents			.15^a^
Still live with parents	50 (96.15%)	53 (100%)	
≤16-year-old	2 (3.85%)	0 (0%)	
Family spending (per month per person) - 1 USD = Rp.15.000			.15^b^
<Rp. 354.000	1 (1.2%)	1 (1.89%)	
Rp. 354.000 – Rp. 532.000	8 (15.38%)	7 (13.20%)	
Rp. 532.000 – Rp. 1.200.000	27 (51.92%)	20 (37.73%)	
Rp. 1.200.000 – Rp. 6.000.000	16 (30.78%)	25 (47.18%)	
>Rp. 6.000.000	0 (0%)	0 (0%)	

^a^ Mann-Whitney test.

^b^ Chi-square.

From the baseline data from the pre-test questionnaires, there was no significant difference in biological parameters (cortisol and alpha-amylase), as well as of the primary (CD-RISC) psychosocial parameter and the secondary psychosocial parameters (knowledge, attitude and behavior, stress perception, DASS score) between subjects in the intervention group and the control group. The initial profile of the participants in the intervention and control groups can be seen in Table 3. Only knowledge and DASS-Anxiety variables were normally distributed, presented as mean, and analyzed using t-test; other variables were not normally distributed, presented as median and analyzed with non-parametric test (Mann Whitney test).

**Table 3:** Baseline pre-test measured outcomes.

Measured baseline	Intervention Group (*n* = 52)	Control Group (*n* = 53)	*P*
	Score	Score	
Resilience (CD-RISC), median (range)	30.00 (19–39)	30.00 (15–40)	.90^a^
Knowledge, mean (SD)	7.12 (1.28)	7.19 (1.46)	.63^b^
Attitudes and behaviors, median (range)	49.00 (40–59)	50.00 (41–58)	.32^a^
Stress perception (PSS), median (range)	18.00 (8–26)	19.00 (4–30)	.17^a^
Depression (DASS-Depression), median (range)	2.50 (0–14)	2.00 (0–12)	.90^a^
Anxiety (DASS-Anxiety), mean (SD)	7.00 (3.89)	5.92 (3.28)	.13^b^
Stress (DASS-Stress), median (range)	9.00 (1–17)	9.00 (1–20)	.80^a^
DASS-Total, median (range)	18.00 (3–41)	18.00 (1–43)	.43^a^
Cortisol S1, ng/ml, median (range)	4.25 (1.21–24.96)	5.69 (0.19–20.94)	.76^a^
Cortisol S2, ng/ml, median (range)	5.36 (0.99–18.68)	4.74 (0.00–22.64)	.19^a^
Delta Cortisol ng/ml, median (range)	−0.03 (−14.70–16.81)	−0.95 (−11.97–17.51)	.15^a^
Alpha-Amylase S1, U/mL, median (range)	105.08 (2.21–354.20)	101.99 (2.18–1,006.80)	.87^a^
Alpha-Amylase S2, U/mL, median (range)	114.18 (10.43–1,200.00)	110.44 (1.67–1,545.60)	.99^a^
Delta Alpha-Amylase, U/mL, median (range)	5.38 (−163.94–1,195.28)	3.33 (−318.72–1,543.42)	.68^a^

^a^ Mann-Whitney non-parametric test.

^b^ t-test.

### 3.2. Primary outcome

**Figure 3:**
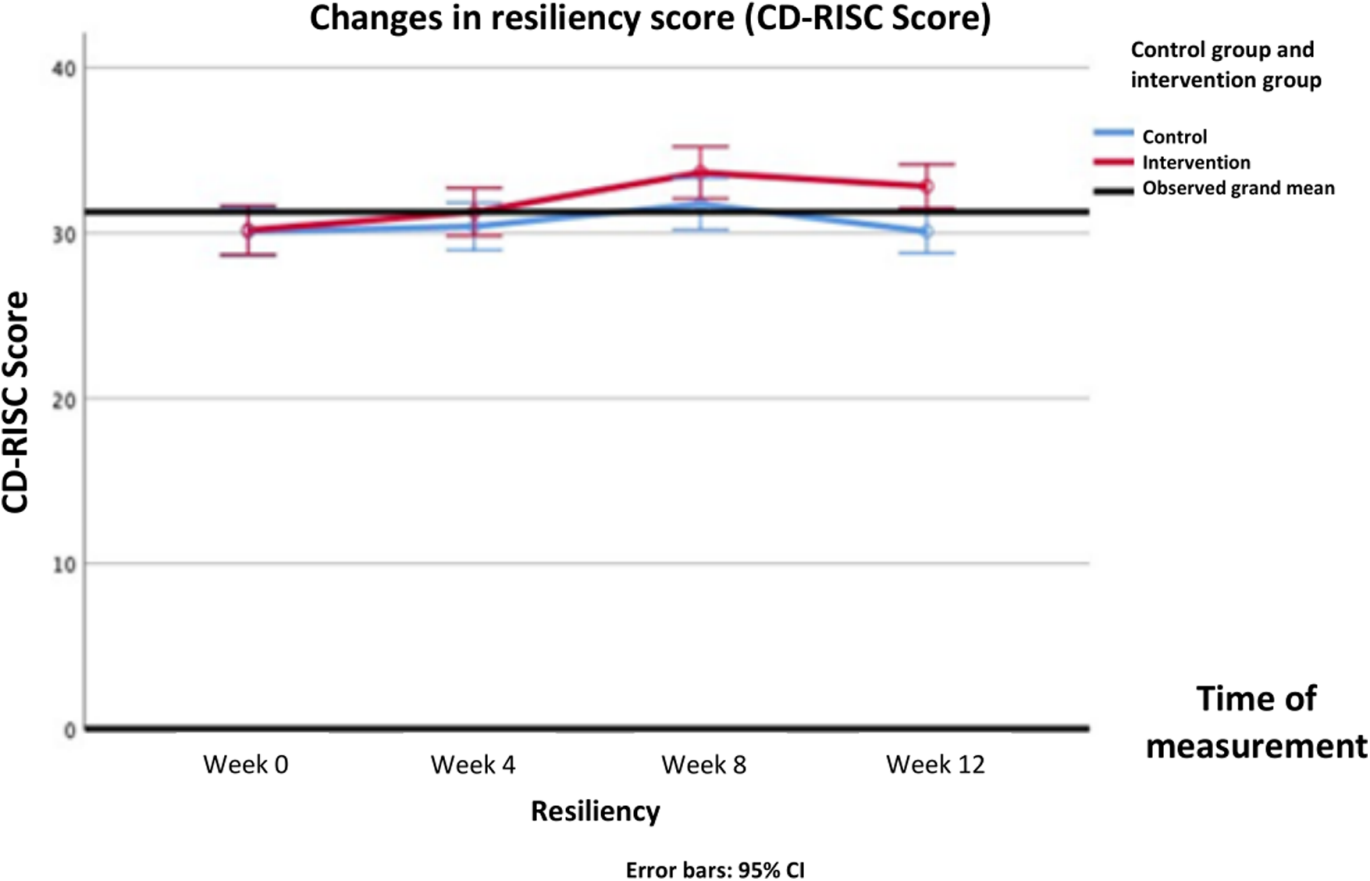
Changes in resiliency score over time (CD-RISC score).

Normality test showed that the data had a normal distribution (*P* > .05), thus parametric tests were used. Analysis of CD-RISC scores of group main effect using repeated measures ANOVA revealed there was no significant increase in the resilience score in the intervention group (*Δ* mean [95%CI] = 1.40 [−0.45–3.26]; F(1, 103) = 2.243, *P* = .137) compared to the control group. However, significant main effect of time [F(3, 309) = 18.191, *P* < .001] and interaction effect between intervention and time [F(3, 309) = 5.056, *P* = .002] was observed in the CD-RISC scores. Bonferroni post-hoc analyses of CD-RISC score differences between group in each time-point showed significant difference in week 12 (*Δ* mean [95%CI] = 2.73 (0.87—4.59); *P* = .004). The changes in CD-RISC scores from the pre-test to the 12-week follow-up in both groups can be seen in Figure 3 and Table 4.

**Table 4:** Comparison of the psychosocial outcomes of the participants during the pre-test and post-test.

Outcomes	Intervention Group		Control Group		Mean differences between group (95% CI)	Group main effect, *P*	Time main effect, *P*	Interaction effect, *P*
	(*n* = 52)		(*n* = 53)					
	Mean	SD	Mean	SD				
Resilience (CD-RISC)					1.40 (−0.45–3.26)	.137^a^	<.001^a^	.002^a^
Pre-test (Week 0)	30.17	5.00	30.08	5.77				
Week 4	31.27	4.67	30.40	5.79				
Week 8	33.65	4.80	31.75	6.48				
Week 12	32.83	4.09	30.09	5.42				
Knowledge					1.73 (1.31–2.15)	<.001^a^	<.001^a^	<.001^a^
Pre-test (Week 0)	7.12	1.28	7.19	1.46				
Week 4	9.13	1.66	7.19	1.46				
Week 8	10.38	1.22	8.53	1.80				
Week 12	10.52	1.48	7.32	1.45				
Attitudes and behavior					1.88 (0.24–3.51)	.025^a^	<.001^a^	<.001^a^
Pre-test (Week 0)	48.50	4.57	49.36	4.28				
Week 4	49.81	4.75	49.15	5.83				
Week 8	53.87	4.08	49.34	6.05				
Week 12	53.13	4.06	49.94	5.03				
Stress perception					−3.41 (−4.70 to −2.12)	<.001^a^	.001^a^	<.012^a^
Pre-test (Week 0)	18.29	4.42	19.64	5.47				
Week 4	17.21	3.99	19.58	5.49				
Week 8	14.96	4.56	19.74	5.52				
Week 12	14.25	4.27	19.40	5.54				
DASS-Depression					−0.95 (−2.09–0.19)	.100^a^	<.001^a^	<.001^a^
Pre-test (Week 0)	3.56	3.43	3.45	3.12				
Week 4	3.31	3.36	3.62	3.30				
Week 8	2.15	3.26	4.04	3.52				
Week 12	1.73	2.58	3.45	3.48				
DASS-Anxiety					−0.67 (−0.51–1.86)	.263^a^	<.001^a^	<.001^a^
Pre-test (Week 0)	7.00	3.89	5.92	3.28				
Week 4	5.08	3.71	5.72	3.07				
Week 8	4.69	3.95	5.64	3.39				
Week 12	3.73	3.23	5.91	3.08				
DASS-Stress					−1.36 (−2.94–0.22)	.091^a^	<.001^a^	<.001^a^
Pre-test (Week 0)	8.58	3.65	8.38	4.47				
Week 4	7.79	3.98	8.64	4.94				
Week 8	6.37	4.53	8.70	4.51				
Week 12	5.65	4.47	8.11	4.73				
DASS-Total					−2.83 (−6.18–0.53)	.098^a^	<.001^a^	<.001^a^
Pre-test (Week 0)	19.13	8.93	17.79	8.54				
Week 4	16.17	9.83	17.98	9.29				
Week 8	13.21	10.37	18.38	9.56				
Week 12	11.12	8.51	16.79	9.65				

^a^ Repeated measures ANOVA.

### 3.3. Secondary outcomes

**Figure 4:**
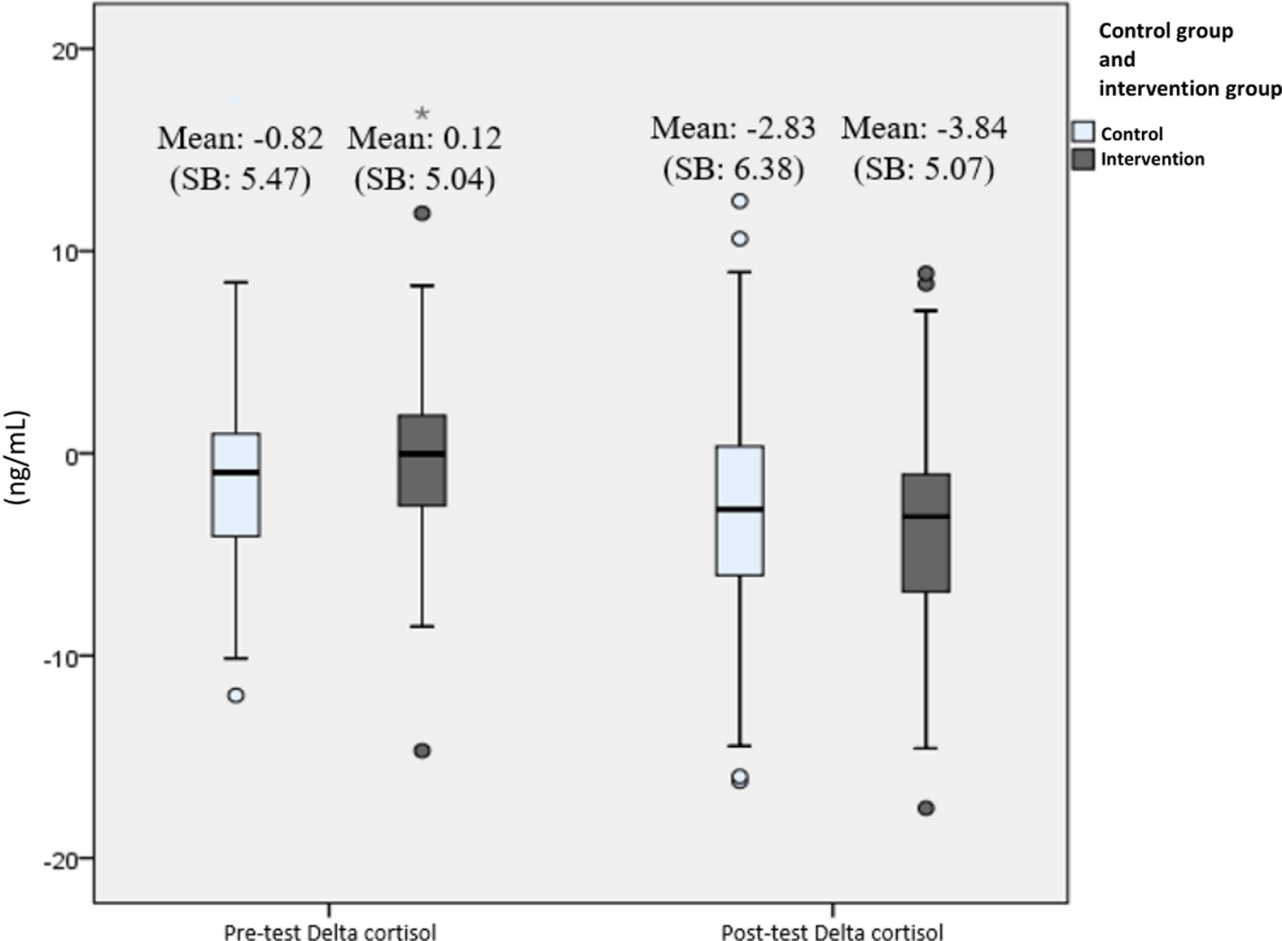
Changes in delta cortisol level (ng/mL) from pre-test to post-test presented with boxplot. The bars represent median and interquartile range, while the tip of the bar represents minimum and maximum value. The spheres represent mild outliers (Q1 – 1.5 * IQR or Q3 + 1.5 * IQR), while the asterisk represent extreme outliers (Q1–3 * IQR or Q3 + 3 * IQR).

**Figure 5:**
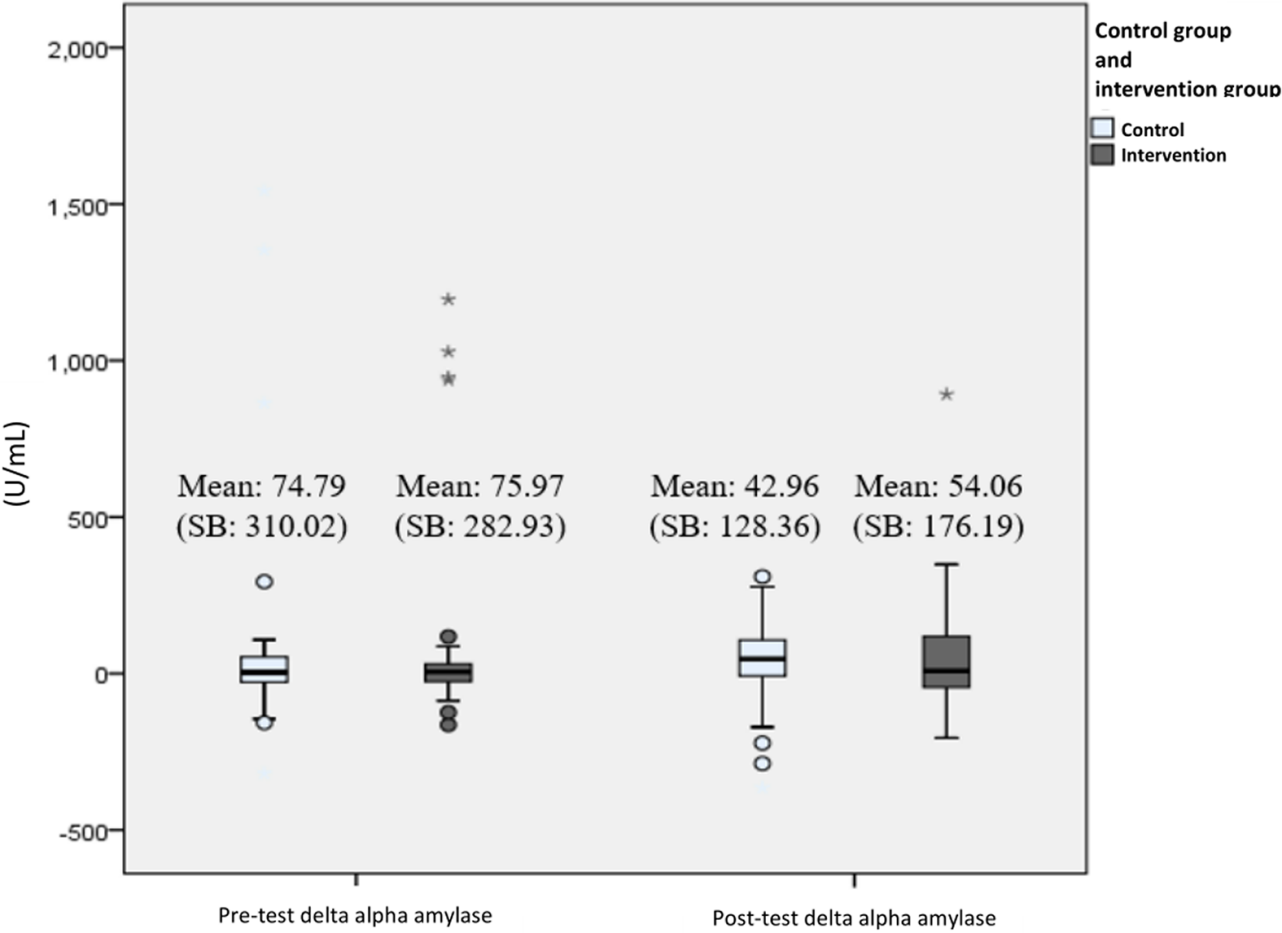
Changes in delta alpha amylase level (U/mL) from pre-test to post-test presented with boxplot. The bars represent median and interquartile range, while the tip of the bar represents minimum and maximum value. The spheres represent mild outliers (Q1–1.5 * IQR or Q3 + 1.5 * IQR), while the asterisk represent extreme outliers (Q1–3 * IQR or Q3 + 3 * IQR).

In all secondary outcome measures, normality tests were conducted and showed that the distribution were normal (*P* > .05), thus all data are presented in mean and parametric tests were used. Biological parameter analysis of the participants' salivary cortisol and alpha-amylase levels showed there was a significant difference in the mean cortisol delta during the pre-test (Mean 0.12) and post-test (Mean – 3.84) at week 12 (*P* < .001) in the intervention group. While for the control group, there was no difference in the mean cortisol delta pre-test (Mean −0.82) and post-test (Mean −2.83) (*P* = .07). For the measurement of alpha-amylase delta biomarker levels, there was no significant difference in the mean at the pre-test and after the 12th week, both in the intervention and control groups. Changes in delta levels of cortisol and alpha-amylase biomarkers during the pre-test and post-test at week 12 can be seen in Figures 4, 5.

For the psychosocial parameters, analysis using repeated measures ANOVA showed main effects of group on knowledge (*Δ* mean [95%CI] = 1.73 (1.31—2.15); F(1, 103) = 66.805, *P* < .001), attitudes and behavior (*Δ* mean [95%CI] = 1.88 (0.24—3.51); F(1, 103) = 5.191, *P* = .025), and stress perception (*Δ* mean [95%CI]=−3.41 (−4.70 to −2.12); F(1, 103) = 27.567, *P* < .001). Main effects of time were also found on all psychosocial parameters: knowledge [F(3, 309) = 76.459, *P* < .001], attitudes and behavior [F(3, 309) = 24.908, *P* < .001], stress perception [F(3, 309)= 5.561, *P* = .005], DASS-Anxiety [F(3, 309) = 13.729, *P* < .001], DASS-Depression [F(3, 309) = 7.018, *P* < .001], DASS-Stress [F(3, 309) = 13.729, *P* < .001], and DASS-Total [F(3, 309) = 23.515, *P* < .001].

Interaction effects between intervention and time were found on all secondary psychosocial outcomes: knowledge [F(3, 309) = 83.700, *P* < .001], attitudes and behavior [F(3, 309) = 36.214, *P* < .001], stress perception [F(3, 309)= 6.970, *P* < .001], DASS-Anxiety [F(3, 309) = 33.384, *P* < .001], DASS-Depression [F(3, 309) = 65.223, *P* = .012], DASS-Stress [F(3, 309) = 26.595, *P* < .001], and DASS-Total [F(3, 309) = 39.229, *P* < .001]. In post-hoc analysis, knowledge scores started to improve significantly after 4th week and forth, while attitude and behavior began to improve after 8th week and forth. Stress perception began to decrease in the 4th week. Mental health problems assessed by DASS questionnaire, namely depression symptoms and stress improved after 8th week, whereas anxiety symptoms improved after 12th week. The DASS-Total score improved after 8th week. All Bonferroni post-hoc analysis of all the secondary psychosocial outcomes score differences between groups in each time point are presented in Table 5.

**Table 5:** Post-hoc analysis of the repeated measures ANOVA of mean score differences between intervention group and control in each time points.

Outcomes	Mean differences between group (95% CI)	*P* ^a^
Resilience (CD-RISC)		
Pre-test (Week 0)	0.10 (−1.99—2.19)	.926
Week 4	0.87 (−1.17–2.91)	.398
Week 8	1.90 (−0.31–4.11)	.091
Week 12	2.73 (0.87–4.59)	.004
Knowledge		
Pre-test (Week 0)	0.07 (−0.46–0.60)	.785
Week 4	1.95 (1.34–2.55)	<.001
Week 8	1.86 (1.26–2.45)	<.001
Week 12	3.20 (2.63–3.77)	<.001
Attitudes and behavior		
Pre-test (Week 0)	0.86 (−0.86—2.57)	.323
Week 4	0.66 (−1.41–2.72)	.529
Week 8	4.52 (2.52–6.53)	<.001
Week 12	3.19 (1.42–4.96)	.001
Stress perception		
Pre-test (Week 0)	−1.35 (−3.28–0.57)	.167
Week 4	−2.37 (−4.24 to −0.51)	.013
Week 8	−4.77 (−6.73 to −2.81)	<.001
Week 12	−5.14 (−7.06 to −3.23)	<.001
DASS-Depression		
Pre-test (Week 0)	0.11 (−1.16–1.37)	.870
Week 4	−0.32 (−1.61–0.98)	.629
Week 8	−1.88 (−3.20 to −0.57)	.005
Week 12	−1.72 (−2.91 to −0.54)	.005
DASS-Anxiety		
Pre-test (Week 0)	1.08 (−0.32–2.47)	.128
Week 4	−0.64 (−1.96–0.68)	.337
Week 8	−0.95 (−2.38–0.48)	.190
Week 12	−2.18 (−3.40 to −0.95)	.001
DASS-Stress		
Pre-test (Week 0)	0.20 (−1.38–1.78)	.803
Week 4	−0.85 (−2.59–0.89)	.333
Week 8	−2.33 (−4.08 to −0.58)	.009
Week 12	−2.50 (−4.24 to −0.68)	.007
DASS-Total		
Pre-test (Week 0)	1.34 (−2.04–4.72)	.433
Week 4	−1.81 (−5.51–1.89)	.335
Week 8	−5.17 (−9.03 to −1.31)	.009
Week 12	−5.68 (−9.20 to −2.15)	.002

^a^ Bonferroni post-hoc analysis.

**Figure 6:**
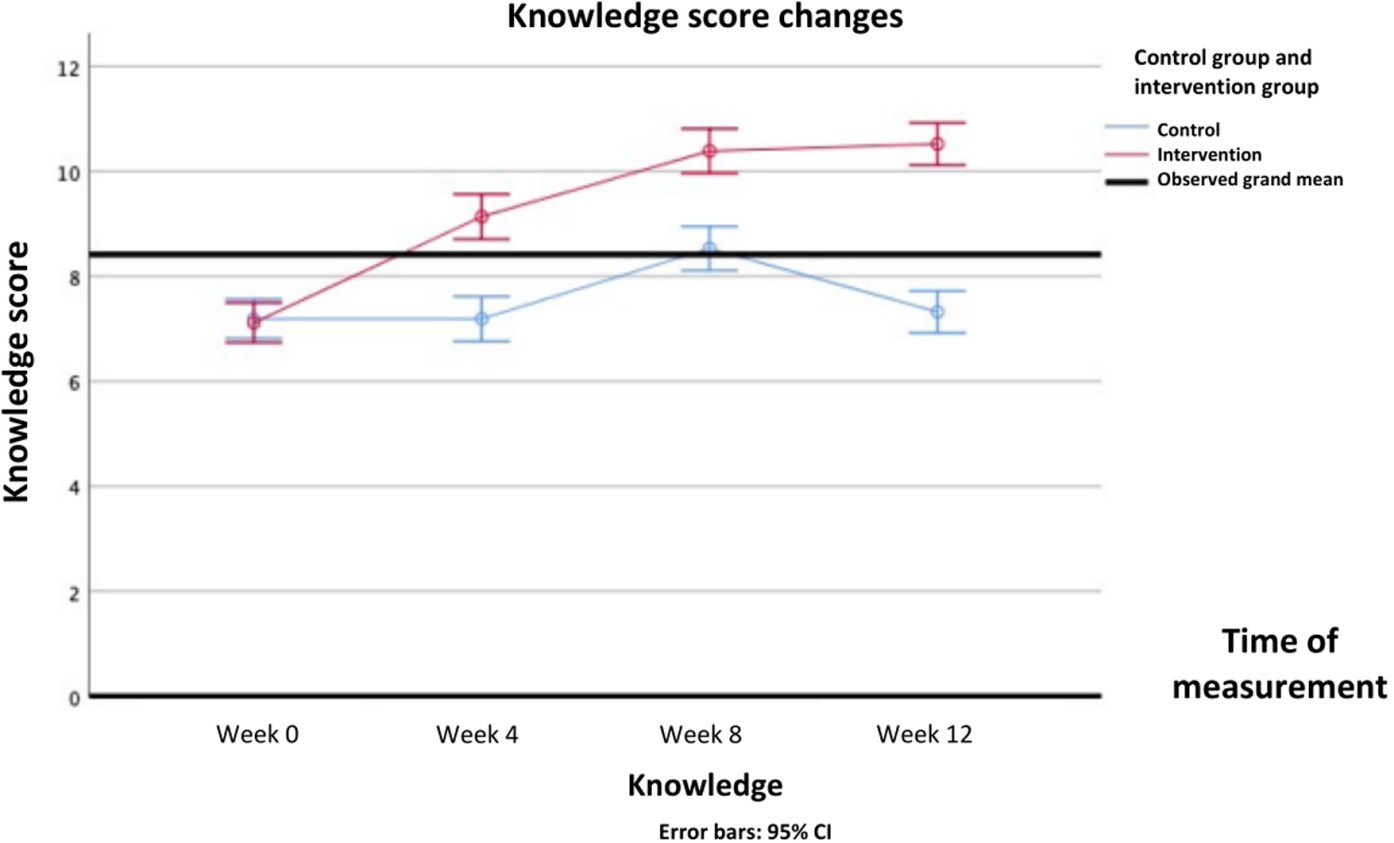
Changes in mental health knowledge score overtime.

**Figure 7:**
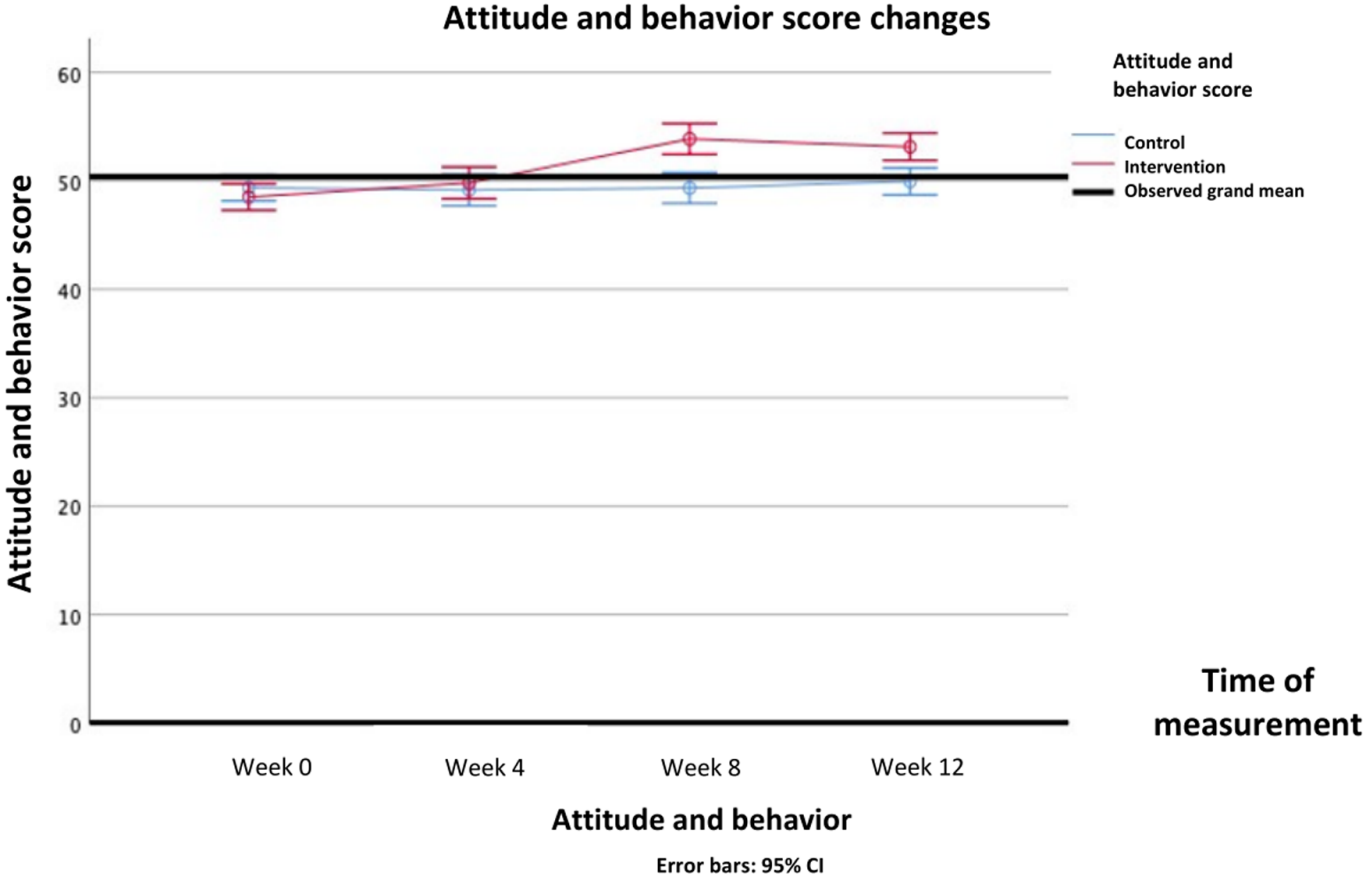
Changes in attitude and behavior score overtime.

**Figure 8:**
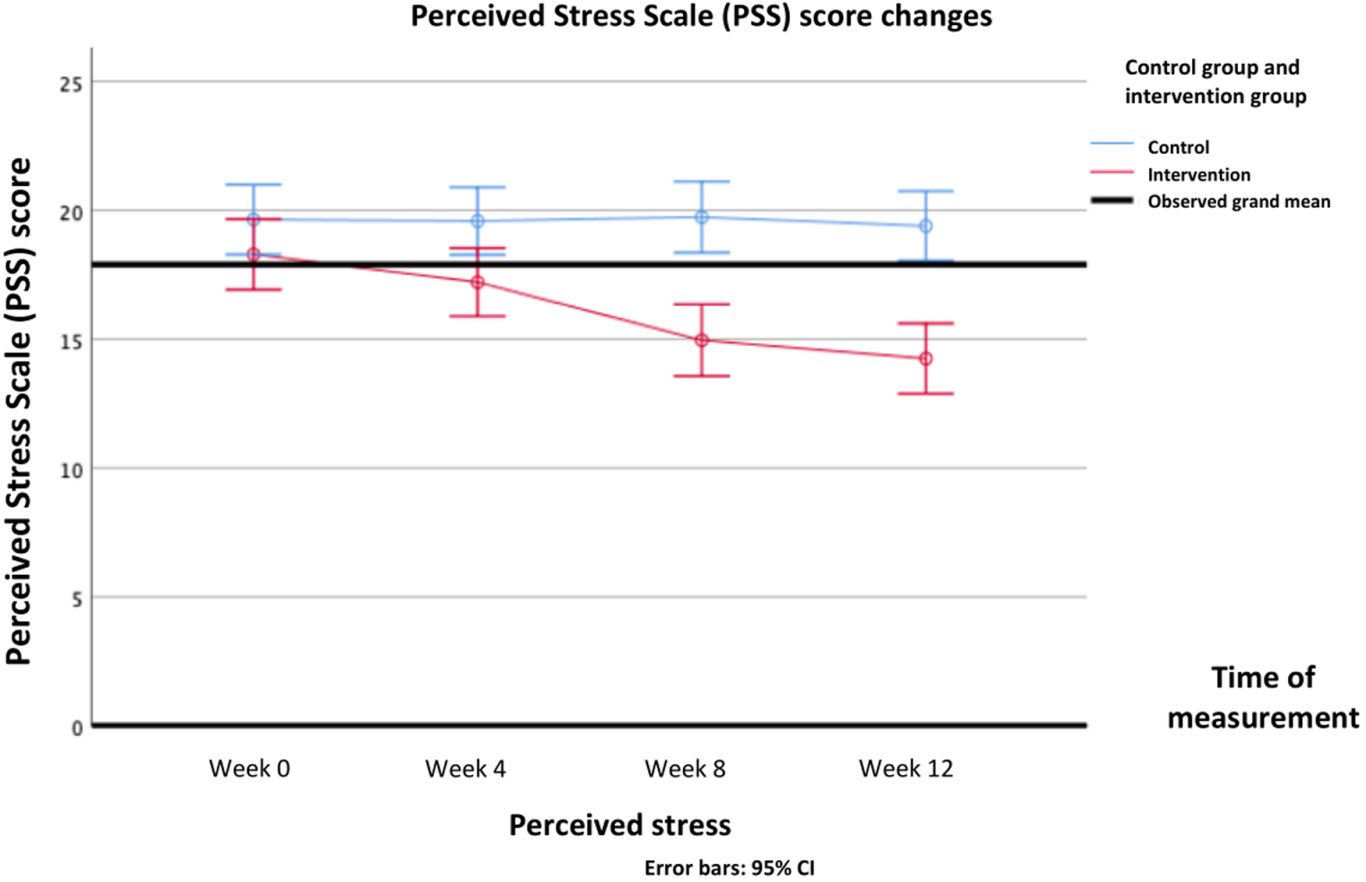
Changes in perceived stress scale (PSS) score overtime.

**Figure 9:**
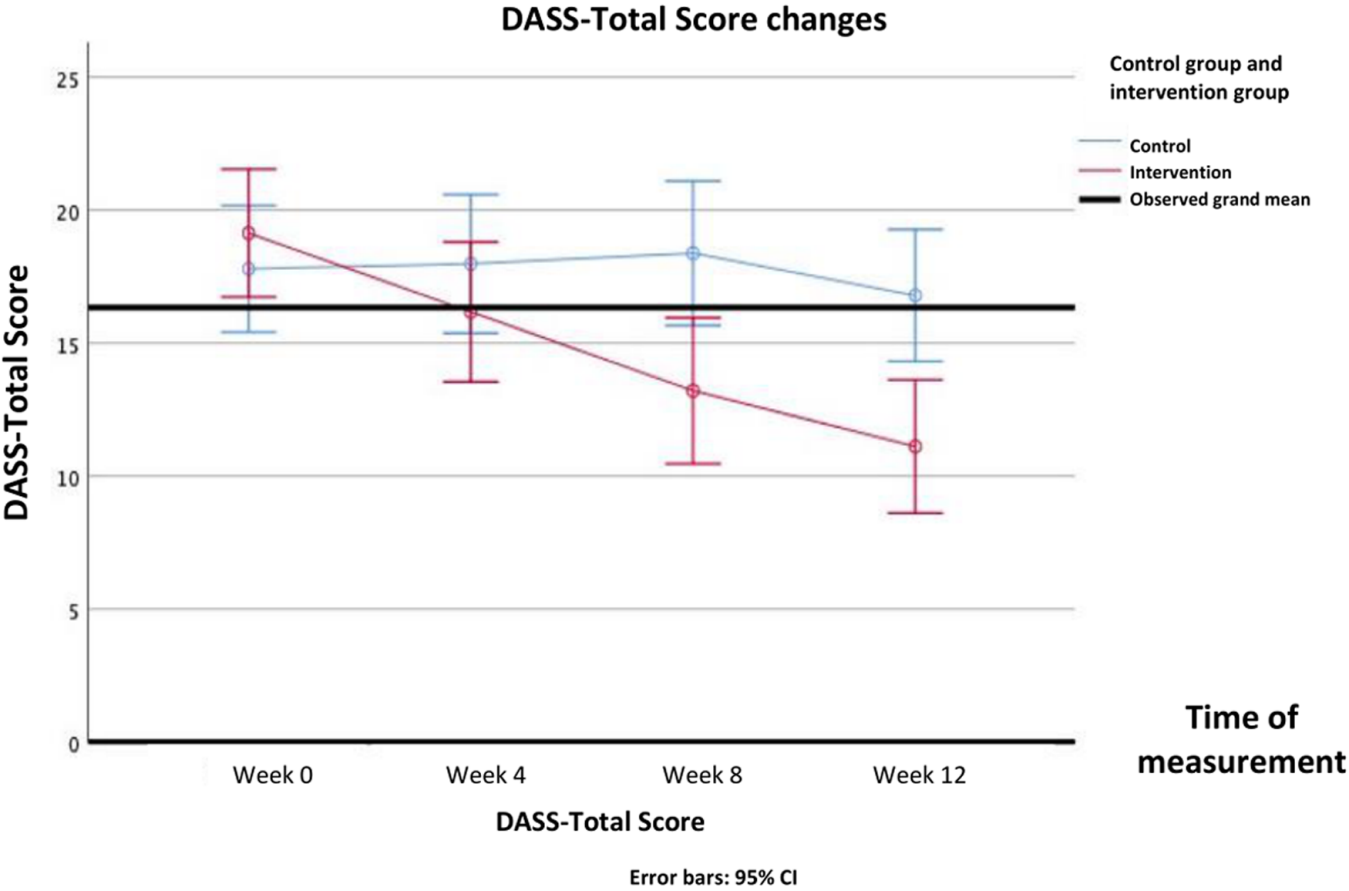
Changes in DASS-total score overtime.

The differences in all resiliency and psychosocial outcomes in the pre-test and post-test in the intervention group and the control group over repeated measurements can be seen in Table 4. The psychosocial profiles of the two groups during the pre-test up to week 12 can be seen in detail in Figures 6–9.

## 4. Discussion

### 4.1. Principal results

Improvement in resilience and all secondary psychosocial outcomes (knowledge, attitude and behavior on mental health; stress perception; symptoms of depression, anxiety, and stress from DASS questionnaire; and the DASS-total score) on the intervention group compared to the control group after certain time points. The resilience score between groups was not found to be significantly improved until week 8 and is found to be significantly improved on week 12. All the secondary psychosocial outcomes had a significant improvement between groups by week 12, while almost all secondary psychosocial outcomes (except anxiety symptoms) also had a significant improvement by week 8. This finding implies that the effectiveness of this intervention to improve resilience may be seen after application of the module for a certain duration, which is similar to previous studies.

Two studies by Roig et al. (21) and Sood et al. (35) also showed an increase in resilience eight weeks after intervention. Zare et al. (36), where a 60–90-minute mental health module course set for over six weeks successfully increased knowledge, reduced stigmatization towards mental disorders and encouraged strategies for self-help under challenging situations. Attitude and behavior improvement toward mental health was also observed in Milin et al. (37) and Ventieri et al. (38); however, both studies were conducted on school students. Regarding symptoms of anxiety, depression, and stress, findings in this study were also supported by an RCT in Malaysia by Mohammadzadeh et al. (39), which found that a mental health coaching intervention in teenagers succeeded in reducing the level of depression, anxiety, and stress.

This study also analyzed the change in salivary cortisol levels as a secondary outcome, and it was found that the cortisol levels were significantly reduced after the intervention. This finding showed that it is biologically evident that subjects who received mental health strengthening module intervention had better emotion regulation and perception control of stress elicited by TSST. Other studies also found a significant reduction in salivary cortisol levels after TSST following intervention (40, 41). It is already widely known that cortisol levels are correlated with stress, which activates the hypothalamic-pituitary-adrenal (HPA) axis that eventually secretes cortisol in humans.

However, this study found no significant difference between salivary alpha-amylase levels after the intervention in both groups. The results of available studies regarding the correlation of alpha-amylase with resiliency were still mixed (42–46). Although alpha-amylase is a specific biomarker of stress, many factors might interfere with its secretion, such as decreased saliva production and increased protein levels due to food intake.

This study is the first to develop a mental health strengthening module for transitional-age youths in the first year of medical school. This module effectively increases resilience to stress measured from various biopsychosocial aspects, such as cortisol, resilience, knowledge, attitude and behavior, perception of stress, and symptoms of depression, anxiety, and stress. One similar study which developed an intensive mental health education module for the first-year medical student was conducted in China. The module was done over an 8-week interval with 90 min sessions per week, resulting in a significant decline in psychological distress and academic burnout while increasing life satisfaction levels (47). This study showed that even with a shorter duration (4 weeks), the module could achieve a similar outcome compared to a longer duration module.

### 4.2. Limitations

This study did not limit the stress exposure experienced by students during the academic schedule while being enrolled in this study. Additionally, by the 12th week of the study, all first-year students were in the final week exam, which might influence the result of the stress biomarker baseline. However, the study outcomes were more contextualized by letting students follow the natural course of university students and take exams as usual. They were closer to the actual setting in which students undergo the adaptation process and face stressors in the university.

## 5. Conclusion

The four-week online mental health strengthening module was found to have significant effects in improving the resilience of medical students, particularly at the end of the study (week 12), and improving various other psychosocial parameters. The positive results of the intervention require 4–12 weeks to take effect in resilience and all secondary psychosocial parameters. Salivary cortisol levels as a stress biomarker were significantly reduced following the intervention.

In order to maintain the sustainability of this module's effectiveness, it is highly advisable to have a program aiming to increase knowledge and skills to overcome mental health problems throughout the medical school program. It is essential to disseminate any information regarding help-seeking behavior for students to be more independent without experiencing stigma. In addition, students can be more sensitive to mental health problems, signs, and symptoms encountered by their friends. Thus, they can be more assistive and follow the procedures of seeking help following the module.

## Data Availability

The data analyzed in this study is subject to the following licenses/restrictions: The datasets presented in this article are not readily available because participants of this study did not agree for their data to be shared publicly. Requests to access further information about the study should be directed to FK, fransiska.kaligis@ui.ac.id.
