# Supplementary material for: Effectiveness of an online mental health strengthening module to build resilience and overcome stress for transitional aged medical students
Source: Front Digit Health. 2023 Oct 4;5:1207583. doi: 10.3389/fdgth.2023.1207583 (PMC10582941; doi:10.3389/fdgth.2023.1207583)
Supplement: Supplementary file 1 [file Datasheet1.pdf]

MODULE CONTENTS LIST

4	21	All	24	Language
LESSONS	VIDEOS	SKILL LEVEL	QUIZ	LANGUAGE

UNDER MODULE 1

UNDER MODULE 2

Stress and Ways to Overcome Stress

GO TO

UNDER MODULE 3

Mental Health Problems and Symptoms of Mental Disorders

GO TO

UNDER MODULE 4

Please fill out the following form if you have any questions.

No

Email

Regarding

Question

SEND

Contact:

Transition Module Research Team

Division of Child and Adolescent Psychiatry

Department of Psychiatry
medical School
University of Indonesia

Address:

Faculty of Medicine, University of Indonesia
Jl. Salemba Raya No. 6 Jakarta 10430

Email:

admin@modultransisi.id

UNIVERSITAS INDONESIA

© 2023 The Transition Module Team.
