# Supplementary material for: Effectiveness of an online mental health strengthening module to build resilience and overcome stress for transitional aged medical students
Source: Front Digit Health. 2023 Oct 4;5:1207583. doi: 10.3389/fdgth.2023.1207583 (PMC10582941; doi:10.3389/fdgth.2023.1207583)
Supplement: Supplementary file 2 [file Datasheet2.pdf]

Sub Module 2

STRESS AND WAYS TO OVERCOME STRESS

Healthy soul, stress and how to deal with stress

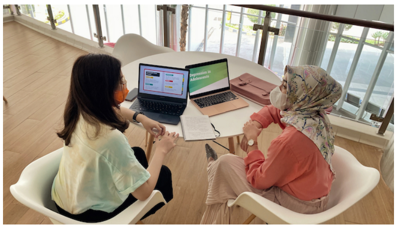

Healthy soul

The definition of mental health according to WHO is:

- Able to accept self and others as they are
- Able to express feelings and have a positive self-image
- Able to withstand the pressures of everyday life
- Able to do daily activities properly, such as work or study
- Able to establish relationships with others and have a sense of caring for others
- Contribute as part of the community

Mental health is an integral part of our physical health and affects our well-being. Mental health or good mental health helps a person to feel and enjoy life positively.

Mental health conditions can change and develop in line with the phases of our lives. Mental health is not a permanent thing, but is formed throughout the process of growth and development of one's personal life.

Having good mental health doesn't mean you never feel sad, upset, angry, or other negative emotions. Often these negative emotions are a sign of our normal mental health.

Many factors affect mental health

- Faktor individu: kepribadian, kemampuan menyesuaikan diri, kepercayaan diri.
- Faktor sosial dan interaksi: keluarga, hubungan dengan orang lain, pekerjaan, komunitas mahasiswa.
- Faktor kemasyarakatan: lingkungan sosial masyarakat yang lebih luas dan politik.
- Faktor budaya: nilai-nilai sosial yang berlaku dan ide-ide tentang kesehatan jiwa.
- Faktor biologis

Mempraktikkan berbagai keterampilan untuk meningkatkan kesehatan jiwa sangatlah berguna. Pilihan dan tindakan kita sehari-hari seperti: berolahraga, bersosialisasi dengan teman, melakukan kegiatan yang menyenangkan, atau melakukan hal-hal baru dapat memengaruhi kesehatan jiwa kita.

Beberapa tautan dan situs untuk mengetahui hal-hal terkait kesehatan jiwa:

- Sehatkah Jiwa Anda? (Kemenkes)
- Beberapa Gejala Tidak Sehat Jiwa (Kemenkes)
- Pengenalan dengan Dokter Jiwa (PDSKJI)
- Mencegah Depresi di Usia Belasan dan Dua Puluhan (PDSKJI)
- Swaperiksa Gejala dan Risiko Depresi, Kecemasan, Bunuh Diri (PDSKJI)
- Video penjelasan ADHD dari Persatuan Dokter Spesialis Kesehatan Jiwa Indonesia (PDSKJI)

Panduan untuk meningkatkan kesehatan jiwa

Panduan berikut bermanfaat untuk meningkatkan kesehatan jiwamu.

- Tidur yang cukup. Kebutuhan tidur sangat berbeda tergantung masing-masing individu, tetapi rata-rata seseorang membutuhkan sekitar delapan sampai sembilan jam tidur setiap malam. Sangat penting untuk memastikan kamu tidur cukup, karena kurang tidur dapat membuatmu mengalami berbagai gejala psikologis, seperti kelelahan, kurang bersemangat, lesu.
- Diet sehat. Pola makan yang bervariasi antara karbohidrat, protein dan sayur-buah serta makan secara teratur akan memberi tubuhmu "bahan bakar" untuk melawan stres. Makanan siap saji atau *junkfood* sebaiknya tidak dikonsumsi terlalu sering.
- Kegiatan di luar ruangan. Berada di dalam ruangan terus menerus akan terasa melelahkan. Kegiatan di luar ruangan, terutama ketika mendapat sinar matahari akan baik untukmu.
- Kegiatan yang menyenangkan dan bermakna. Melakukan hal menyenangkan dengan orang-orang yang membuatmu bahagia akan memberikan pengaruh baik bagimu.
- Setidaknya setengah jam beraktivitas fisik harian, seperti berjalan, dianjurkan untuk melepaskan hormon endorphin yang meningkatkan rasa senang.
- Isolasi sosial tidaklah baik. Meskipun kamu tidak suka bepergian keluar rumah bersama orang lain, namun berinteraksi dengan orang lain bisa membuat kamu merasa lebih baik.
- Pemecahan masalah. Pikirkan satu masalah pada satu waktu dan pikirkan solusi yang mungkin dilakukan. Minta bantuan orang yang kamu percaya. Jangan mencoba untuk mengatasi sendiri semua masalah sekaligus.
- Berbicara dan menulis. Berbagi perasaan dengan orang-orang terkasih yang dipercaya, akan mengurangi beban dan kekhawatiran. Terkadang menulis juga membantu kamu mengurai permasalahan sehingga menjadi lebih jelas.
- Latihan berpikir. Bayangkan situasi yang menantang atau menegangkan dalam pikiran kamu. Pikirkan situasinya langkah demi langkah dan bayangkan bagaimana kamu bisa melewatinya. Ulangi latihan ini beberapa kali. Kamu akan menemukan bahwa meskipun situasinya sulit, kamu akan dapat mengatasinya.
- Mengidentifikasi pikiran negatif. Belajarlah untuk mengenali, menguji, dan mengelola pikiran negatif kamu. Ketika muncul pikiran negatif, berhentilah sejenak, periksa kembali apakah hal tersebut rasional atau ada buktinya, kemudian carilah hal-hal yang lebih positif. Belajarlah untuk mengenali hal-hal yang lebih rasional dan apabila kamu berhasil memperkuat pikiranmu yang lebih rasional, beri apresiasi pada dirimu.
- Sebaiknya luangkan waktu sehari-hari untuk melatih relaksasi. Yoga, meditasi, dan latihan pernapasan adalah cara yang baik untuk bersantai.
- Hitung anugerahmu. Setiap orang pernah mengalami kejadian buruk dalam hidupnya, namun hal ini tidak akan berlangsung terus menerus. Tulislah hal-hal baik yang pernah kamu alami dalam hidup – buat daftarnya dan lihatlah setiap kali kamu mengalami stres.
- Perkuatlah spiritualitasmu. Mengambil waktu untuk berdoa dan beribadah sering kali membantu kita menjadi lebih damai dalam menghadapi tantangan dan masalah.
- Menghindari zat psikoaktif atau adiktif. Meskipun alkohol dan banyak obat-obatan dapat membuat masalah terasa lebih mudah untuk sementara waktu, namun demikian dalam jangka panjang justru akan memperburuk gejala-gejala depresi.
- Jadwal dan rutinitas harian. Rencanakan hari-harimu. Carilah kegiatan untuk dilakukan, mulailah dengan hal-hal sederhana. Bahkan hal-hal kecil seperti mandi, berjalan, atau makan siang bisa membuatmu merasa lebih baik. Rutinitas harian memiliki dampak besar pada suasana hatimu. Menulis buku harian atau jadwal dapat membantumu, terutama jika kamu seorang yang lebih suka hal-hal visual.

Gangguan jiwa dan masalah kesehatan jiwa

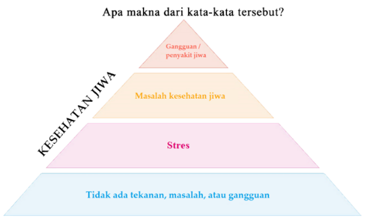

Stres, masalah kesehatan jiwa dan gangguan jiwa semuanya memiliki arti yang berbeda (lihat piramida di atas).

Satu kondisi dalam piramida ini tidak selalu mengarah ke kondisi yang lain, meskipun dapat juga terjadi demikian. Seseorang mungkin untuk mengalami kondisi stres, masalah kesehatan jiwa dan gangguan jiwa secara bersamaan atau bergantian.

Stres itu dapat terjadi pada semua orang – bahkan dapat terjadi setiap hari. Stres atau mengalami tekanan bukanlah suatu penyakit. Pengobatan tidak diperlukan untuk kondisi orang yang mengalami stres. Kondisi ini adalah sinyal bahwa kamu perlu menyelesaikan masalah yang menyebabkan munculnya kesulitan; jadi kamu akan beradaptasi dengan mengubah diri sendiri atau lingkunganmu. Misalnya: terlambat untuk ikut kelas pagi, hal itu merupakan tekanan bagimu sehingga kamu mengalami stres. Dari kejadian tersebut kamu akan belajar untuk menghadapi kejadian selanjutnya dan beradaptasi dengan kondisi tadi, misalnya dengan cara memilih naik kendaraan umum daripada berjalan kaki ketika waktu masuk masuk kelas tinggal sebentar lagi, atau mungkin juga kamu perlu bangun setengah jam lebih awal.

Masalah kesehatan jiwa adalah masalah yang bermakna pada emosi, pikiran, atau perilaku (atau ketiganya) yang menyebabkan kamu merasa kesulitan dalam menghadapi tantangan hidup.

Biasanya situasi ini akan mengharuskan kamu untuk mendapatkan bantuan dari teman, keluarga, orang yang kamu percayai atau seorang profesional, misalnya konselor. Contohnya adalah kesedihan yang kamu alami jika seseorang yang dekat denganmu meninggal, atau periode penyesuaian setelah pindah ke tempat yang sebelumnya tidak dikenal.

Masalah kesehatan jiwa bukanlah penyakit. Ini adalah tanda bahwa kamu mengalami kesulitan beradaptasi, tetapi hal itu wajar terjadi mengingat terdapatnya stressor yang kamu hadapi. Kamu tidak memerlukan pengobatan medis untuk masalah kesehatan jiwa ini, namun memerlukan perhatian dan dukungan dari orang lain untuk sementara waktu.

Gangguan jiwa. Kondisi ini dapat terjadi pada siapa saja dan terjadi di manapun di seluruh dunia. Sekitar 10-20% anak dan remaja di bawah usia 21 tahun mengalami gangguan jiwa pada periode tertentu di kehidupan mereka dan jumlahnya terus bertambah seiring bertambahnya usia. Kesadaran masyarakat tentang gangguan jiwa saat ini berada pada tingkat yang cukup tinggi; Namun, sering kali kesadaran ini tidak selalu disertai dengan pengetahuan dan pemahaman. Orang mengetahui gangguan jiwa bisa menjadi masalah – tetapi banyak orang tidak mengerti apa sebenarnya gangguan jiwa itu.

Sulit untuk membedakan antara gejala gangguan jiwa dan kesulitan mengendalikan emosi dan pikiran yang umum terjadi dalam kehidupan sehari-hari. Transisi menuju kehidupan mandiri memiliki sangat banyak tantangan dan menjadikan periode ini lebih berisiko untuk mengalami gangguan jiwa. Penting untuk memahami apa itu kesehatan jiwa dan apa itu gangguan jiwa, serta apa yang perlu dilakukan jika kamu mencurigai dirimu (atau temanmu) mungkin mengalami gangguan jiwa (untuk informasi yang lebih terperinci tentang masalah kesehatan jiwa dan gangguan jiwa, lihat sub tema 3).

Mental Health Literacy Pyramid Explained (Explication de la pyramide de litarcie en santé mentale)

Stres

Memahami dan mengelola respons stres

Kata "stres" yang sering kita ucapkan sebetulnya adalah kependekan dari "respon stres", yang merupakan cara otak dan tubuhmu memberi tahu, bahwa kamu memiliki tantangan atau masalah yang perlu ditangani. Ini adalah sinyal untuk proses adaptasi dan merupakan dasar untuk membangun ketahanan. Akan tetapi "stres" saat ini telah berkonotasi negatif yang mengarah pada cara berpikir yang salah dalam mengelola respon stres kita.

Menggunakan bahasa yang jelas untuk menggambarkan apa yang terjadi akan membantu kita belajar bagaimana menggunakan respon stres untuk meningkatkan kesehatan jiwa kita. Orang juga sering mengganti kata "kecemasan" ketika mereka merujuk pada respon stres; Namun kecemasan tidak sama dengan respon stres, karena kecemasan merupakan keadaan khawatir dan waspada berlebih yang terus menerus.

Mitos stres jahat

Sampai saat ini kebanyakan orang percaya bahwa respon stres itu buruk bagi kita dan oleh karena itu, kapan pun harus dihindari. Namun sebagian besar stres yang kita alami setiap hari sebenarnya baik untuk kita, bahkan dengan menghindarinya justru akan menjadi kurang baik. Kuncinya adalah kita tidak mengurangi jumlah stres yang dialami, tetapi mempelajari bagaimana cara terbaik untuk menghadapi stres yang muncul dalam kehidupan.

Kurva respon stres

Respon stres dapat dikelola dengan cara yang sehat, yang dapat mendukung proses beradaptasi, dan membantu membangun ketahanan mental kita. Diagram di bawah ini menunjukkan kurva respon stres harian yang khas.

Ketika menghadapi stresor (misalnya ujian, mendapat perlakuan tidak menyenangkan dari kelompok, mendapat nilai buruk, bertengkar dengan teman atau pasangan), otak dan tubuhmu akan merespon dengan sinyal yang memberi tahu bahwa kamu memiliki masalah yang perlu diselesaikan. Kurva respon stres akan naik, memuncak, dan kemudian secara bertahap akan menurun. Pada kurva tersebut, ada tiga poin manajemen stres, masing-masing perlu digunakan untuk membantumu mengelola situasi dengan baik.

Stress (Le stress)

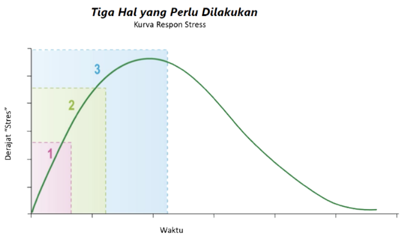

1. Pada titik ini, fokusnya adalah pada **bagaimana kamu berpikir tentang pengalaman yang kamu anggap sebagai stres**. Penelitian menunjukkan bahwa jika kita melihat sinyal ini sebagai negatif (seperti: Saya merasa stres; Saya tidak bisa menangani ini; Saya merasa kewalahan), respon tubuh kita menjadi tidak sehat, mengarahkan kita untuk menghindari situasi atau mengharapkan orang lain untuk menyelesaikan masalah untuk kita. Namun, jika kita melihat sinyal ini sebagai sesuatu yang positif (seperti: Saya ditantang untuk menyelesaikan masalah; otak dan tubuh saya bersiap-siap untuk membantu saya mengatasi tantangan ini; Ini hanya sementara, saya akan belajar banyak hal setelah melewati ini), respon tubuh kita menjadi lebih sehat, membuat kita bersiap untuk beradaptasi dan berubah. Kondisi yang seperti ini merupakan respon stres yang diharapkan.
2. Pada titik ini, fokusnya adalah pada **menentukan apa permasalahnya dan mengembangkan solusi untuk menyelesaikannya**. Misalnya: respon stres karena harus mengerjakan tugas yang banyak sekali sebelum ujian, akan mengarahkanmu mengembangkan keterampilan cara belajar atau bertemu dengan dosen, kakak tingkat atau teman lain untuk membahas beberapa konsep yang tidak sepenuhnya kamu pahami. Ketika kamu memecahkan masalah yang telah diidentifikasi oleh respon stres, masalah tersebut akan hilang dan kamu akan memiliki keterampilan baru yang dapat kamu gunakan di masa depan ketika menghadapi kondisi yang mirip. Dengan demikian, kamu telah berhasil beradaptasi.
3. Pada titik ini, fokusnya adalah pada **intensitas respons stres kamu**. Kadang-kadang respon stres kita bisa terasa sangat intens (terutama jika pola perilaku kita sebelumnya sering kali menghindari stres) dan pada titik ini, teknik-teknik yang untuk mengurangi respon stres atau manajemen stres akan bermanfaat.

▸ **resilience**

▸ **Cigarettes, alcohol and psychoactive substances are not suitable for stress management**

- Gambling and other behavioral addictions
- Mindfulness

QUIZ 2

MODULE LIST

Contact:

Transition Module Research Team

Division of Child and Adolescent Psychiatry

Department of Psychiatry
medical School
University of Indonesia

Address:

Faculty of Medicine, University of Indonesia
Jl. Salemba Raya No. 6 Jakarta 10430

E-mail:

admin@modultransisi.id

UNIVERSITAS INDONESIA

© 2023 The Transition Module Team.
