# Supplementary material for: Effectiveness of an online mental health strengthening module to build resilience and overcome stress for transitional aged medical students
Source: Front Digit Health. 2023 Oct 4;5:1207583. doi: 10.3389/fdgth.2023.1207583 (PMC10582941; doi:10.3389/fdgth.2023.1207583)
Supplement: Supplementary file 3 [file Datasheet3.pdf]

Indonesian → English

UNIVERSITAS INDONESIA

HOME. ABOUT CONTACT LOGIN

TRANSITION AND ADAPTATION TO RESILIENCE

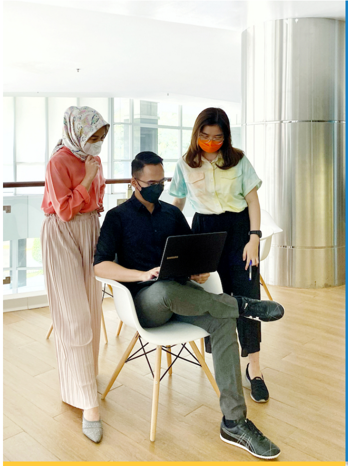

Coping With Life Changes, Growing Up And Becoming Resilient

Recognizing Stress and Strengthening Mental Health Modules in the Adaptation Process of New Students at the Faculty of Medicine.

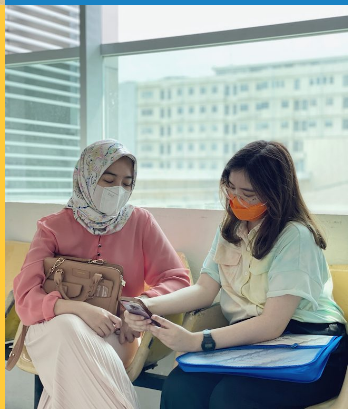

Introduction Videos

Let's watch the following video to find out what Transition is and what challenges first year students will face.

START STUDY

Contact:

Transition Module Research Team
Division of Child and Adolescent Psychiatry
Department of Psychiatry
medical School
University of Indonesia

Address:

Faculty of Medicine, University of Indonesia
Jl. Salemba Raya No. 6 Jakarta 10430

E-mail:

admin@modultransisi.id

UNIVERSITAS INDONESIA

© 2023 The Transition Module Team.
